# The role of social support in reducing the long-term burden of cumulative childhood adversity on adulthood internalising disorder

**DOI:** 10.1007/s00127-024-02674-6

**Published:** 2024-04-30

**Authors:** Mary Buchanan, Giles Newton-Howes, Ruth Cunningham, Geraldine F. H. McLeod, Joseph M. Boden

**Affiliations:** 1 Department of Psychological Medicine, University of Otago, Wellington, New Zealand; 2 Department of Public Health, University of Otago, Wellington, New Zealand; 3 Department of Psychological Medicine, University of Otago, Christchurch, New Zealand

**Keywords:** Childhood adversity, Adverse childhood experiences, Birth cohort, Mental health, Social support

## Abstract

**Purpose:**

Previous research indicates that social support is protective for the mental health outcomes of exposure to childhood adversity. However, the impact of social support as a protective factor following exposure to cumulative childhood adversity is understudied with prospective longitudinal data. The aim of this present study was to examine how social support mediates the impact of cumulative exposure to childhood adversity on internalising disorder in adulthood.

**Methods:**

The Christchurch Health and Development Study (CHDS) is a general population birth cohort, born in 1977 and representative of Christchurch, New Zealand at the time of the cohort members’ birth. The present study used a generalised estimating equations (GEE) framework to analyse direct associations between a cumulative measure of childhood adversity (CA) and internalising disorders (major depression, and any anxiety disorder), and indirect associations through social support.

**Results:**

Results indicated a dose-dependent relationship between increased exposure to CA and worsened odds of a diagnosis for major depression and any anxiety disorder, respectively. There was also a significant mediating effect of social support on the direct associations between CA and both major depression (OR (95%CI) =0 .98 (0.97, 0.99), p < 001) and any anxiety disorder (OR (95%CI) = .98 (0.97, 0.99), p < 001).

**Conclusion:**

The findings indicate that social support reduces the impact of childhood adversity on adult mental health, and is therefore a target for future work examining potential interventions following CA.

**Supplementary Information:**

The online version contains supplementary material available at 10.1007/s00127-024-02674-6.

## Introduction

### Cumulative childhood adversity and internalising disorder

Childhood adversity (CA) refers to experiences that pose a threat to a child’s physical or psychological wellbeing. It is common, with most estimates from Western developed countries indicating that over a half of children have experienced at least one type of adversity [1–3]. Childhood adversities include a broad range of factors including abuse, household dysfunction, social problems, financial hardship, and parent instability or mental health problems [4, 5]. CA is broadly defined, with terms such as childhood maltreatment [6], childhood trauma [7] and adverse childhood experiences (ACEs) [8] all describing elements of CA.

The wider literature suggests a particularly strong link between CA and internalising disorders in adulthood [9–11], particularly when measured at a population level. A systematic review of prospective studies by Li, D’Arcy and Meng [12] found that childhood maltreatment substantially increases the risk of depression and anxiety in adulthood. Similarly, experiences of CA are frequently reported by adults suffering from mental health disorders [13, 14]. The seminal Kaiser-Permanente ACEs study first described the dose-dependent pattern of the associations between childhood adversity and deleterious outcomes in adulthood, whereby adult outcomes are incrementally worse with increasing exposures to adversity [8]. This pattern has been demonstrated repeatedly in the 20 years of literature since, particularly in relation to mental illness outcomes [14, 15] including anxiety and depression [3, 16]. This literature suggests that the accumulation of instances of exposure to adversity in childhood, rather than the unique contributions of different adversities, predicts adult deleterious outcomes [17], including psychopathology [18]. Therefore, when investigating the population-level relationships between childhood adversities and internalising disorders, it is appropriate to treat adversities as cumulative, particularly when there is a focus on adversity generally, as opposed to specific adversities.

It is important to note that there are issues with predicting individual mental health outcomes based on ACE scores, due to the high variability in presentation of adversities [19]. While the generalised effect of higher accumulation of adversities can be measured, this has only limited clinical utility as a prognostic tool. Additionally, there are ethical concerns around screening for CA, using cumulative scores such as the ACEs score, as it frames children’s experiences through a deficit lens [20].

High levels of CA exposure are associated with significant social and economic costs [21, 22]. CA is considered a leading cause of health inequalities within and between countries with an increased risk for socioeconomically disadvantaged populations [22]. The CDC has made recommendations for prevention of CA, including: strengthening economic supports to families, promoting social norms that protect against violence and adversity, ensuring a good start for children, teaching skills for parents, connecting youth to caring adults and activities, and intervening to lessen harms [23]. Successful prevention programs have shown improvements in parenting practices, socioemotional skills, access to high quality childcare, support for parent mental health and substance use disorders. While prevention should be the first priority, it is not possible to prevent all adversity exposures to children. Additionally, it should be noted that exposure does not deterministically lead to psychopathology, in fact, many thrive in adulthood despite highly adverse childhoods [24]. Therefore, alongside prevention efforts, research has turned to examining factors that promote or enhance positive adaptation despite exposure to CA, thus predicting better mental health than in the absence of such factors.

### The role of social support

Certain internal characteristics have been found to predict improved outcomes following CA, including self-esteem, adaptive cognitive-emotional strategies and self-forgiveness and hope [24], as well as resilience as a characteristic itself [25]. Resilience research has since turned to a more ecological systems approach in which both internal and external factors are deemed important for positive outcomes following adversity [26]. The majority of the research on protective factors focuses on factors that are present before or during the exposure to adversity, and which buffer the acute effect of the adversity. Less well studied are factors that act after the adversity has occurred, and which reduce its ongoing effects later in life.

One important area of research has been social support as a protective factor. Social support is the resources provided by one’s social network intended to benefit their ability to cope with stress [27, 28]. A systematic review [29] found that social support is a key protective factor and is associated with mitigated internalising disorder following CA. An example of the protective role of social support is shown in the reduction of depressive symptoms and is consistent with neuropsychological research demonstrating that social support moderates the effect of stress on the brain [30]. Furthermore, aside from service-based or psychotherapy interventions, social support and social skills training have long been key clinical approaches to reducing negative outcomes following CA [31–33]. Mentoring programs, for example, have especially been widely adopted by governments to improve outcomes for at-risk youth populations, such as the internationally implemented Big Brothers Big Sisters programme [34].

The present study aimed to examine the role of social support as a mediator between cumulative CA and depression and anxiety outcomes in a general population birth cohort. When analysing mediation, longitudinal data is particularly valuable, as it allows for consideration of the temporal sequencing of variables, and is less ambiguous to interpret than concurrent mediation analyses [35]. In recent years, there have been various longitudinal studies of the role of social support as a protective factor [29]. However, these have mostly measured CA retrospectively in adulthood, in some cases, CA has been measures decades after exposure when participants were in their 50s or 60s [36–38]. Retrospective accounts of adversity, and the passage of time between exposure and recall may lead to recall bias in these studies [13]. A substantial proportion of individuals known to have suffered abuse or maltreatment do not report such abuse when interviewed in adult life [39]. Furthermore, adults with psychopathology tend to retrospectively recall more CA than those without psychopathology [40]. This bias could lead to overestimation of the association between CA and internalising disorders. Therefore, CA should be measured prospectively wherever possible, except in cases where the subject matter is not suitable to assess with child participants (e.g. sexual abuse).

Against this background, the present investigation examines the role of social support in mediating the associations between CA and internalizing disorders in adulthood, using data from a longitudinal birth cohort. The present study utilised data from theChristchurch Health and Development Study (CHDS), a prospective birth cohort which was representative of the New Zealand population in the 1970s. The CHDS has measured cumulative CA prospectively from birth throughout childhood and repeatedly measured social support and internalising symptoms to age 40. The current study aims to examine social support as a protective factor between cumulative CA to age 16, and internalising disorders (major depression and anxiety disorder) during adulthood.

## Methods

### Participants

The Christchurch Health and Development Study (CHDS) is a birth cohort of participants studied at repeated intervals throughout their life course. The initial cohort comprised all children born in the Christchurch urban region between 15 April and 5 August 1977. Out of a total of 1310 live births during this period, the parents of 1265 (97%) of infants agreed to participate. Detailed cohort information can be found in published in study profiles: [41–43].

The CHDS is representative of the New Zealand population at the time of birth of the cohort members [44] in terms of demographic measures including biological sex, socioeconomic status (SES) and ethnicity. The cohort at birth was 13% Māori, 3% Pasifika, and 84% New Zealand European. Over time, this distribution has changed. Māori were more likely to be retained in the study, and some members chose to identify as primarily Māori later in life even if previously identified otherwise. At age 40, 17% of the cohort identified as Māori, 3% Pasifika, and 80% New Zealand European.

### Procedures for data collection

Assessments have occurred at birth, 4 months, annual intervals up to age 16 and at ages 18, 21, 25, 30, 35 and 40 years. This cohort has been studied using a combination of: in face-to-face interviews with parents and participants, standardised testing, teacher report and official record data [41, 45]. All study information was collected based on signed informed consent from study participants.

## Measures

### Outcomes

#### Major depression and anxiety disorders

At ages 18, 21, 25, 30, 35 and 40 years cohort members were assessed with an interview that combined relevant components of the Composite International Diagnostic Interview (CIDI) [46] and custom written survey items to assess DSM-IV symptom criteria [47] for major depression and anxiety disorders (generalised anxiety disorder, panic disorders, agoraphobia, social phobia, specific phobia). Participants were classified as meeting criteria for a) major depressive disorder and b) any anxiety disorder for each assessment period at ages 16–18, 18–21, 21–25, 25–30, 30–35, and 35–40 years.

### Predictors

#### Childhood adversity (CA)

A CA score representing exposure to adversity in childhood was constructed in previous analyses from the CHDS data [18]. Five domains comprised of 20 measures were assessed: poverty, parental adjustment problems; family violence; adolescent mental health and psychological problems; and adolescent adjustment problems. Each domain was made up of four measures chosen from the CHDS database, based on previous research, including findings from the CHDS cohort [18]. Details of these measures and the age at which they were assessed are described in Appendix A. Participants were classified as 0 or 1 on each measure representing the presence or absence of each adversity. Participants’ scores on each item were summed to derive a measure of CA representing the number of adversities experienced by each participant before age 16. When the score was developed, the cumulative count score had the strongest predictive effects for adulthood adversity, beyond the sum of its parts. Consistent with previous literature [8, 48], the count score treats all adversity as equal regardless of the nature and timing of the exposures. As adversities tend to co-occur, this approach has higher ecological validity than analysing independent effects of different adversities. These scores were then classified into quintiles for the purposes of data analysis. Widaman and Revelle [49] argue that sum scores perform equally well to factor scores in which a single factor model fits well. Details of this analysis can be found in the original description of this measure [18].

#### Social support

Social support was assessed at ages 18, 21, 25, 30, 35 and 40. Two measures of social support were used. At ages 18, 21 and 25, participants were asked to report the number of female and male friends they had (respectively; a lot, quite a lot, a few, none). The number of friends were averaged across both questions (i.e. male friends and female friends). The reliability of this score, between ages 18 and 25 was α = 0.66, reflecting changes in number of friends over this age period. At ages 30, 35 and 40 participants were asked to identify the number of people that would provide various types of support (none, one, 2–3, 4–5, 6 +). For example, “if you were sick in bed for several weeks, how many people would help you?”, and “how many people do you know whose advice you really trust?”. The reliability for this score, between ages 30 and 40 was α = 0.89. To attain a consistent measurement from age 18 to age 40, the social network size reported at each assessment period were standardised across the six waves of assessments to create a score for each assessment period with a mean of 100 and a standard deviation of 10. There was consistency within individuals over the two scores, suggesting a latent ‘sociability’. The overall alpha reliability for the combined social support score was α = 0.78.

#### Covariates

A wide range of covariate factors were selected from the study database to control the associations in the model for the effects of confounding. These covariates were selected on the basis that they were not included in the CA measure, were associated with CA and were also correlated with the outcomes in the respective models. The following demographic factors were measured at birth: biological sex, ethnic identity, mother’s age, maternal and paternal education, and family SES. The other covariates were: change in family structure from birth to 16 years; child neuroticism and extraversion at age 14; and child novelty seeking at age 16. Detailed descriptions of covariate measures are provided in Appendix B. All covariates were entered into the final model using forward and backward methods of variable entry, to attain stable and parsimonious models.

#### Statistical analysis

Datasets for the present analyses were prepared in SAS 9.4 [50]. All analyses were carried out using Stata 17 [51]. For the following analyses, we used random effects logistic Generalised Estimating Equation (GEE) models, in which repeated measures outcomes are modelled as a linear function of the predictors. GEE regression models permit the repeated measures of each outcome for each individual to be correlated [52, 53].

First, tabular methods were used to test the bivariate associations between CA, social support and internalising disorders. We report the direct associations pooled across time points, and p-values derived from z-statistics. In the Results section, Model 1 describes the unadjusted regression equation of CA on internalising disorders. All analyses include a term for the interaction of CA and time period, as the magnitude of the association between CA and both major depression and any anxiety disorder decreased over the assessment periods from ages 16–18 years to 35–40 years.

Second, the GEE models were extended to include potentially confounding variables. After forwards and backwards selection of covariates, of which the full list is presented in Appendix B, analyses were adjusted in the following way. For the outcome major depression, analyses were adjusted for: biological sex, SES at birth, neuroticism (14 years) and novelty seeking (16 years). For the outcome anxiety disorder, analyses were adjusted for: biological sex and neuroticism (14 years). Model 2 describes the adjusted regression equation of CA and selected covariates on internalising disorders.

Third, the GEE models were extended to examine potential mediation by social support on the direct association between CA and the internalising disorder outcomes. Model 3 describes the adjusted regression equation of CA on internalising disorder outcomes after the addition of the social support measure.

Mediation was ascertained following the Baron and Kenny criteria [54], whereby a given variable may be said to function as a mediator to the extent that it accounts for the association between the predictor and the outcome. This approach requires that when controlling for the bivariate associations with social support, previously significant associations between CA and internalising disorders are no longer significant, or are reduced in magnitude. Therefore, to ascertain mediation, the social support measure was added to the adjusted regression model describing the associations between CA, the covariates and the outcome.

## Results

### Associations between CA and social support in adulthood

Table 1 shows mean social support scores for each quintile of CA at each assessment period at 18, 21, 25, 30, 35 and 40 years. The table also provides a pooled estimate of the mean and standard deviation of social support for each level of CA over the assessment periods from age 18 to age 40, as well as a test of significance for the pooled association between CA and social support as derived from random-effects GEE models.

**Table 1:** Mean (sd) scores on the standardised social support score for each CA quintile at ages 18, 21, 25, 30, 35 and 40 years, pooled across observations

	Cumulative childhood adversity score quintiles				
	1	2	3	4	5
Age (years)	Social support score *M* (sd)				
18	100.41 (10.10)	100.44 (9.44)	100.91 (9.93)	98.56 (10.45)	98.96 (10.82)
21	101.70 (9.53)	100.46 (9.85)	100.36 (9.80)	97.84 (10.20)	97.59 (11.69)
25	102.65 (9.67)	100.58 (9.82)	99.23 (9.43)	96.61 (10.36)	99.20 (10.72)
30	102.10 (9.22)	101.23 (8.38)	99.79 (9.33)	96.55 (11.10)	95.91 (13.35)
35	102.09 (8.84)	101.35 (8.83)	99.82 (8.82)	96.90 (12.20	93.35 (9.87)
40	102.44 (8.77)	101.38 (8.88)	99.00 (8.99)	97.47 (12.13)	91.64 (13.94)
Pooled score	101.88 (9.40)	100.89 (9.22)	99.87 (9.41)	97.32 (11.05)	96.36 (11.97)

Bivariate association between CA and social support, adjusted for confounders: B = − 1.40 (.18), p < .001

The table shows that there was a dose-dependent pattern in the association between increasing levels of CA exposure and lower levels of social support that was consistent across assessment periods. The pooled association was found to be statistically significant (B(SE) = − 1.40 (0.18), p < 0.001). Examination of the pooled scores show that those with the lowest level of CA exposure had the highest average social support score of 101.88 (sd = 9.40), compared to those with the highest exposure who had the lowest average social support score of 96.36 (sd = 96.36).

### Associations between CA and internalising disorder in adulthood

Table 2 shows the frequency of major depression and any anxiety disorder for each quintile of exposure to CA, at each assessment period at 16–18, 18–21, 21–25, 25–30, 30–35 and 35–40 years. The table also provides a pooled estimate of the prevalence of major depression and any anxiety disorder for each level of CA over the assessment periods from 16–18 years to 35–40 years, as well as respective tests of significance of the pooled associations between CA and major depression and any anxiety disorder, after adjusting for covariates, as derived from random effects GEE models.

**Table 2:** Percent meeting criteria for major depression and anxiety disorder, respectively, for each CA quintile at ages 16–18, 18–21, 21–25, 25–30, 30–35 and 35–40, pooled across observations

	Cumulative childhood adversity score (quintiles)				
	1	2	3	4	5
Age (years)	Percent meeting criteria for major depression				
16-18	11.0	13.3	26.6	35.0	65.0
18-21	14.1	21.1	27.8	31.2	53.8
21-25	13.8	16.9	24.1	35.0	48.7
25-30	12.9	19.5	24.2	26.8	44.7
30-35	12.1	14.7	17.7	28.9	52.8
35-40	16.5	15.7	20.0	33.6	58.3
ORs (95%CI)	1	1.5 (1.41, 1.77)	2.49 (1.98, 3.14)	3.94 (2.78, 5.56)	6.21 (3.92, 9.86)

	Cumulative childhood adversity score (quintiles)				
	1	2	3	4	5
Age (years)	Percent meeting criteria for any anxiety disorder				
16-18	7.6	10.8	17.6	31.4	60.0
18-21	5.5	9.5	15.7	19.6	38.5
21-25	5.9	12.9	17.6	27.3	35.9
25-30	11.2	13.9	17.7	25.4	44.7
30-35	9.3	11.6	14.8	27.4	38.9
35-40	13.9	11.7	23.0	29.4	50.0
OR (95%CI)	1	1.53 (1.36, 1.73)	2.35 (1.84, 3.01)	3.61 (2.50, 5.21)	5.54 (3.40, 9.03)

Bivariate association, adjusted for confounders, between CA and major depression,: B = .46 (.06), p < .001; any anxiety disorder: B = .43 (.06), p < .001

Table 2 shows that there was a dose-dependent association between increasing levels of CA and higher prevalence of both major depression and any anxiety disorder that was consistent across all time periods. The adjusted pooled associations were found to be statistically significant (both p < 0.001). Examination of the pooled prevalence shows that those with the highest level of CA exposure had odds of meeting criteria for major depression that were 6.21 times higher than those with the lowest level of exposure. The pooled prevalence also indicate that those with the highest level of CA exposure had odds of meeting criteria for any anxiety disorder that were 5.54 times higher than those with the lowest level of exposure. The strength of the association between CA exposure and major depression weakened over time (B = − 0.05, p = 0.01), as did the strength of the association between CA exposure and any anxiety disorder (B = − 0.06, p = 0.002).

### CA * time period interaction term

In addition, there was a statistically significant interaction term for CA and time period for the models of both major depression and any anxiety disorder (major depression B(SE) = − 0.05 (0.02), p = 0.01); anxiety disorder B(SE) = − 0.06 (0.02) p = 0.002). In both cases, the interaction term suggested that the magnitude of the association between CA and both major depression and any anxiety disorder decreased over the assessment periods from ages 16–18 years to 35–40 years.

### Associations between social support and internalising disorder

Table 3 shows the prevalence of major depression and any anxiety disorder (respectively) for each quintile of the social support score, at each assessment period at 16–18, 18–21, 21–25, 25–30, 30–35, and 35–40 years. The table also provides pooled estimates of the prevalence of major depression and any anxiety disorder, respectively, for each level of social support over the assessment periods from age 16–18 to age 35–40, adjusted for covariates, as derived from random effects GEE models.

**Table 3:** Percent meeting criteria for major depression and any anxiety disorder, respectively, for each social support quintile at ages 16–18, 18–21, 21–25, 25–30, 30–35 and 35–40, pooled across observations

	Social support score (quintiles)				
	1	2	3	4	5
Age (years)	Percent meeting criteria for major depression				
16–18	26.4	21.7	23.5	20.7	14.8
18–21	28.7	28.1	21.9	15.5	18.1
21–25	34.8	27.3	25.0	17.4	11.6
25–30	31.0	18.8	21.6	17.0	17.4
30–35	29.0	15.1	16.0	14.2	18.4
35–40	33.3	20.8	22.0	16.3	13.9
OR (95% CI)	1	0.89 (0.84, 0.95)	0.80 (0.70,0.91)	0.71 (0.59, 0.86)	0.63 (0.49,0.82)

	Social support score (quintiles)				
	1	2	3	4	5
Age (years)	Percent meeting criteria for any anxiety disorder				
16–18	23.3	18.3	14.9	19.2	8.2
18–21	15.6	15.6	12.0	9.0	8.6
21–25	28.3	17.7	16.4	13.0	10.1
25–30	24.5	13.8	17.6	15.2	14.7
30–35	21.7	14.0	13.0	14.7	11.5
35–40	31.5	18.0	20.4	16.3	13.3
OR (95% CI)	1	0.98 (.97, .99)	0.78 (.68, .90)	0.69 (.56, .86)	0.61 (.46, .82)

Bivariate association, adjusted for confounders, between social support and major depression: B = -.02 (.004), p < .001; any anxiety disorder: B = -.02 (.005), p < .001

The table shows that there was an association between increasing levels of social support and lower prevalence of both major depression and any anxiety disorder. The adjusted pooled associations were found to be statistically significant (both p < 0.001). Examination of the pooled prevalence show that those with the highest level of social support had prevalence of major depression that were 37% lower than those with the lowest level of social support. The pooled prevalence also show that those with the highest level of social support had prevalence of any anxiety disorder that were 39% lower than those with the lowest level of social support.

### Regression and mediation analyses

Table 4 shows the results of the three GEE models testing first the association between CA and major depression and any anxiety disorder, second these associations adjusted for confounders, and third the mediating contribution of social support. Table 4 displays odds ratios with 95% confidence intervals representing the odds of major depression and anxiety, respectively, for those with the highest level of CA exposure as compared to those with the lowest level of CA exposure. It also displays the results of tests of significance of the effects. The prevalence of major depression and any anxiety disorder both increase as a function of higher CA exposure, both before and after adjustment for potentially confounding variables (Major Depression: OR (95% CI) = 1.73 (1.56, 1.91) and 1.59 (1.42, 1.78) respectively, both p < 0.001; any Anxiety Disorder: OR (95% CI) = 1.87 (1.67, 2.09) and 1.65 (1.47, 1.84) respectively, both p < 0.001).

**Table 4:** Odds of internalising disorders (Major depression and any anxiety disorder) in a birth cohort associated with increasing levels of cumulative childhood adversity: crude (Model 1), adjusted for confounders (Model 2) and further adjusted for social support (model 3)

Major depression			
	Model 1	Model 2	Model 3
	OR (95% CI)		
Exposures			
Cumulative childhood adversity	1.73 (1.56, 1.91)***	1.59 (1.42, 1.78)***	1.55 (1.39, 1.74)***
Social Support			0.98 (.97, .99)***
CA x Time Period	0.96 (.92, .99)**	0.95 (.91, .98)**	0.95 (.90, .98)**
Covariates			
Biological sex		1.99 (1.57, 2.53)***	1.97 (1.55, 2.50)***
Father’s education		1.29 (1.10, 1.52)**	1.31 (1.12, 1.54)**
Neuroticism		1.07 (1.04, 1.10)***	1.07 (1.04, 1.10)***
Novelty seeking		1.03 (1.00, 1.05)*	1.03 (1.00, 1.05)*

Any anxiety disorder			
	Model 1	Model 2	Model 3
Exposures			
Cumulative childhood adversity	1.87 (1.67, 2.09)***	1.65 (1.47, 1.84)***	1.60 (1.43, 1.79)***
Social Support			0.98 (.97, .99)***
CA x Time Period	0.95 (.91, .98)**	0.94 (.90, .98)**	0.94 (.90, .98)**
Covariates			
Biological sex		2.15 (1.66, 2.80)***	2.13 (1.64, 2.76)***
Neuroticism		1.08 (1.04, 1.17)**	1.08 (1.04, 1.16)***

*p < .05, **p < .01 ***p < .001

To test whether social support mediated the adjusted association between CA and major depression and any anxiety disorder, Model 3 was extended to include the time dynamic measure of social support (18, 21, 25, 30, 35 and 40 years) to the terms included in Model 2. The inclusion of social support (Model 3) reduced the magnitude of the association between CA and both major depression, and any anxiety disorder, but these associations remained statistically significant (Major depression: OR (95% CI) = 1.55 (1.39, 1.74), p < 001; any anxiety disorder: OR (95% CI) = 1.60 (1.43, 1.79) p < 0.001). Social support had a significant, negative association with major depression (OR (95% CI) = 0.98 (0.97, 0.99), p < 001) and any anxiety disorder (OR (95% CI) = 0.98 (0.97, 0.99), p < 001) after adjusting for CA and other covariates. This pattern of results suggests that social support played a weak but detectable mediating role in the associations between CA and internalising disorder.

## Discussion

The present study aimed to examine the role of social support as a protective factor between cumulative CA and internalising disorders in adulthood. This is the first study of which the authors are aware to examine this effect using prospectively measured cumulative CA in a general population cohort.

There was a dose-dependent association between an accumulation of exposures to CA and increased odds of meeting criteria for major depression and anxiety disorders in adulthood. also found that social support plays a statistically significant but relatively weak role in reducing the magnitude of the relationship between CA and both major depression and any anxiety disorder in adulthood. This finding is consistent with the wider literature detailing the protective role of social support for mental health, particularly in adolescence and young adulthood, and for those who have experienced adversity in childhood [36, 55–57]. Therefore, the findings of the present study provide evidence to the idea that support from a network of friends and family may protect against the development of internalising disorder in those with histories of high levels of exposure CA [29]

The strengths of the present study overcome limitations present in previous studies of the protective effect of social support. First, the GEE framework enabled the utilisation of repeated measures of social support and internalising disorders to capitalize upon the richness of the available data and increased power. Second, the present study included control for confounding by several factors, which was possible due to the measurement of a wide range of variables from birth in the CHDS [43]. The variables included were measured contemporaneously to the variables that constituted the CA measure [18], therefore controlling more accurately the potentially confounding effects. Third, prospective measurement of CA is a key strength of the present study. The only exception was for abuse variables, which were validated with repeated measurements (age 18 and 21) [58]. Very few longitudinal studies of social support as a protective factor for internalising outcomes of CA have measured CA prospectively [29]. The current study therefore addressed an important gap in the literature by avoiding a large amount of the recall bias inherent in previous studies.

Although this study used birth cohort data, which provides some of the most rigorous data within observational research [59], the study has its limitations. The CHDS cohort was representative of the NZ population at the time of the cohort’s birth, which is now over four decades ago, and as population demographics change over time, the cohort necessarily becomes less representative. This study represents the sociocultural context in which it was conducted, specific to both place and time. Similarly, a limitation of the present study is the use of measures that are, largely superseded. The social support measure used in the later waves (age 30–40 years) is more precise than the measure used in the earlier waves (age 18–25 years) because it accounts for the different ways in which friends provide support, compared to a measure of the size of one’s social network. Unfortunately, this is a common limitation of longitudinal studies, as they rely on what was relevant at the time of measurement. This limitation is offset considerably by the benefits of having repeated measures data and control of within-subjects variability.

Future studies should consider the relative influence of different aspects of social support. This may include the timing, the source (e.g. from family, friends, spouse), and the nature of the support (quality versus quantity). Though this study analysed the contribution of social support with repeated measures over time, it was not able to examine the particular influence of social support at different ages. Research suggests that social support may be particularly beneficial at times of transition, such as the transition from high school into employment or tertiary education [60]. Therefore, a potential avenue for future research is to examine whether the presence of social support is particularly protective at these times. Further, some studies point to the role of different sources of support, and that support from family may be most influential [36, 61]. They also suggest distinguishing quality and quantity of social support [37, 61], though the effect may be strongest when both are enhanced [56]. Examination of these distinctions with prospective cohort data could elucidate further details of how social support promotes resilience.

The findings of the present study provide empirical support for interventions based upon enhancing social support to improve internalising outcomes. This finding does, however, suggest that in common practice and discourse, the effect of social support may be overestimated. The small, though detectable, effect size observed indicates that social support alone is unlikely to exert enough difference to be clinically important. One explanation for the comparably convincing impact of social-support-based interventions is that enhancing social support has effects beyond increasing the size of the social network. For example, successful interventions may also drive self-esteem building, and psychoeducation [62]. The combined effect of these factors requires investigation. The lack of strong effect sizes in this area of research suggests that when directing intervention efforts, the prevention of exposure to CA in the first place remains key.

This paper provides evidence that social support plays a weak but detectable role in mitigating major depression and anxiety disorders following CA. It suggests that while CA has a concerning association with adult internalising disorders, it is not deterministic. Social support may be one driver of positive outcomes following CA, but it may not be enough on its own. On a population level these findings do not suggest that there would be many detectable improvements in mental illness outcomes if social support alone were enhanced. The findings of the present study suggest that social support needs to be researched further to determine how it acts alongside other factors to improve internalising disorder outcomes. Social support is a key target for strategies that will reduce the long-term burden of CA, but this study also highlights the fundamental importance of preventing CA.

## Supplementary Information

Below is the link to the electronic supplementary material.

Supplementary file1 (DOCX 54 KB)
