# Supplementary material for: The role of social support in reducing the long-term burden of cumulative childhood adversity on adulthood internalising disorder
Source: Soc Psychiatry Psychiatr Epidemiol. 2024 Apr 30;59(12):2165–75. doi: 10.1007/s00127-024-02674-6 (PMC11522040; doi:10.1007/s00127-024-02674-6)
Supplement: Supplementary file 1 — Supplementary file1 (DOCX 54 KB) [file 127_2024_2674_MOESM1_ESM.docx]

Appendix A – Cumulative childhood adversity

**Poverty**

Measures included: averaged family living standards (0-10 years) assessed on the basis of annual interviewer ratings, averaged family income (0-10 years and 11-14 years), and number of years of family welfare dependence (0-14 years).

**Parental adjustment problems**

Measures included: parental history of illicit substance use (11 years), parental history of criminal offending, alcohol problems, or depression/anxiety (15 years).

**Family violence**

Measures included: childhood physical punishment (<16 years), childhood sexual abuse (<16 years), parental intimate partner violence (<16 years) [1], and parental overprotection and control (<16 years) using the Parental bonding Instrument (PBI) [2].

**Adolescent mental health and psychological problems**

Measures included: major depression and anxiety using the relevant (self-or parent-report) Diagnostic Interview Schedule for Children (DISC) [3], suicidal ideation/attempt (14-16 years) [4], and low self-esteem (15 years) [5].

**Adolescent adjustment problems**

Measures included: conduct/oppositional defiant disorder (14-16 years) assessed using the parental interview from the Revised Behaviour Problems Checklist [6], and the Self-Report Early Delinquency Scale (SRED) [7]; participants were assessed using the SRED and the Diagnostic Interview Schedule for Children (DISC) [7]. Alcohol abuse (14-16 years) was assessed using a questionnaire based on Casswell, Stewart, Connolly and Silva [8] and a modified version of the Rutgers Alcohol Problem Index [9]. Questions on cannabis use (14-16 years) and contact with Police (14-16 years) were custom written for the study.

Appendix B - Covariate Measures

**Demographics at birth**

At the first wave of data collection, when the cohort were 0 months old, their biological sex, ethnic identity and mother’s age were measured. Biological sex was classified as male or female. Ethnicity was dichotomised to form a score representing Māori or non-Māori identity. Maternal age at first birth was assessed in whole years at the time of the cohort member’s birth.

Maternal and paternal education were assessed at the time of the child’s birth using 3-point scales that reflected the mother’s and father’s respective highest level of educational attainment. The scales were as follows: 1, mother/father lacked educational qualifications; 2, mother/father had secondary (high school) qualifications; and 3, mother/father had tertiary (college) qualifications.

**Change in family structure**

Comprehensive data on the child’s family placement and changes of parents were collected at annual intervals from birth to age 16 years. To assess the extent of parental change, a measure of the child’s exposure to parental change was constructed by counting the number of changes of parent(s) from birth to 16 years.

**Child personality**

Child personality was assessed at age 14 years using the short form of the Eysenck Personality Inventory (EPI) [10] . This measure comprises two subscales of neuroticism (10 items) and extroversion (10 items). Items were scored on a 3-point scale (not like me/ a bit like me/ a lot like me). Total scale scores for neuroticism and extroversion were constructed based on a sum of the items in each subscale, scored such that higher scores implied higher neuroticism or extroversion. The two scales were of moderate internal consistency (α=0.80, 0.83 respectively).

**Child Novelty Seeking**

At age 16, cohort members were assessed on the novelty seeking scale of the Tridimensional Personality Questionnaire [11]. This 31-item scale provides a measure of the extent to an individual considered themselves to be “impulsive, exploratory, excitable, disorderly and distractible'' [11] ( p.411). Scale items were summed to produce an overall novelty-seeking measure. Higher scores on the scale reflect higher self-esteem. The internal consistency of the scale was α=0.76.

Appendix C – Table of covariates

| Covariate | Correlation with CA |
| --- | --- |
| Biological sex | .07 |
| Māori at birth | -.23 |
| Mother’s age | -.31 |
| Mother’s education | -.31 |
| Father’s education | -.27 |
| Change in family structure | .50 |
| Neuroticism at 14 | .31 |
| Extraversion at 14 | .05 |
| Novelty seeking at 16 | .23 |

Appendix D - Sample size and sample bias

The present analyses were based on samples of 1,025 at age 18, 1,011 at age 21, 1,004 at age 25, 987 at age 30, 962 at age 35, and 902 at age 40, representing 74% to 82% of the surviving cohort at each age. To examine the effects of sample loss on the representativeness of the sample, the obtained samples with complete data at each age were compared with the remaining sample members on sociodemographic measures collected at birth. This analysis suggested statistically significant (p<.01) tendencies for the obtained samples to underrepresent individuals from socially disadvantaged backgrounds characterized by low parental education, low socioeconomic status, and single parenthood. To address this issue, data-weighting methods were used to examine possible implications of selection effects arising from the pattern of missing data [12]. These analyses produced essentially the same pattern of results as those reported here, suggesting that the conclusions of this study were unlikely to have been influenced by selection bias.

References

[1] M. A. Straus, “Measuring Intrafamily Conflict and Violence: The Conflict Tactics (CT) Scales,” 1979. [Online]. Available: https://www.jstor.org/stable/351733

[2] G. Parker, H. Tupling, and L. B. Brown, “A Parental Bonding Instrument,” *British Journal of Medical Psychology*, vol. 52, no. 1, pp. 1–10, 1979, doi: 10.1111/j.2044-8341.1979.tb02487.x.

[3] A. J. Costello, C. Edelbrock, R. Kalas, M. Kessler, and S. A. Klaric, “The National Institute of Mental Health diagnostic interview schedule for children (DISC) Rockville,” *MD: National Institute of Mental Health*, 1982.

[4] D. M. Fergusson, J. L. Horwood, and M. T. Lynskey, “Prevalence and Comorbidity of  DSM-III-R  Diagnoses in a Birth Cohort of 15 Year Olds,” *J Am Acad Child Adolesc Psychiatry*, pp. 1127–1134, 1993.

[5] S. Coopersmith, *SEI - Self Esteem Inventories*. Palo Alto, CA: Consulting Psychologists Press, 1981.

[6] H. C. Quay and D. R. Peterson, *Manual for the Revised Behaviour Problem Checklist*. Miami: H.C. Quay & D.R. Peterson, 1987.

[7] T. E. Moffittt and P. A. Silvat, “SELF-REPORTED DELINQUENCY: RESULTS FROM AN INSTRUMENT FOR NEW ZEALAND*,” 1988.

[8] S. Casswell, J. Stewart, G. Connolly, and P. Silva, “A longitudinal study of New Zealand children’s experience with alcohol,” *Br J Addict*, vol. 86, no. 3, pp. 277–285, 1991, doi: 10.1111/j.1360-0443.1991.tb01780.x.

[9] H. White and E. W. Labouvie, “Towards the assessment of adolescent problem drinking,” *J Stud Alcohol*, vol. 50, no. 1, pp. 30–37, 1989, doi: 10.15288/jsa.1989.50.30.

[10] H. J. Eysenck and S. B. G. Eysenck, *Manual of the Eysenck Personality inventory.* Hodder & Stoughton, 1964.

[11] C. R. Cloninger, “A Systematic Method for Clinical Description and Classification of Personality Variants A Proposal.” [Online]. Available: https://jamanetwork.com/

[12] J. B. Carlin, J. Carlin, R. Wolfe, C. Coffey, and G. C. Patton, “TUTORIAL IN BIOSTATISTICS ANALYSIS OF BINARY OUTCOMES IN LONGITUDINAL STUDIES USING WEIGHTED ESTIMATING EQUATIONS AND DISCRETE-TIME SURVIVAL METHODS: PREVALENCE AND INCIDENCE OF SMOKING IN AN ADOLESCENT COHORT,” *STATISTICS IN MEDICINE Statist. Med*, vol. 18, pp. 2655–2679, 1998, doi: 10.1002/(SICI)1097-0258(19991015)18:19<2655::AID-SIM202>3.0.CO;2.
